# Editorial: The path towards precision health: Prospects and challenges

**DOI:** 10.3389/fmed.2022.989746

**Published:** 2022-10-11

**Authors:** Muntaser E. Ibrahim, Ayman Hussein, Maria G. Stathopoulou, Sophie Visvikis-Siest

**Affiliations:** ^1^Department of Molecular Biology Institute of Endemic Diseases, University of Khartoum, Khartoum, Sudan; ^2^Institut National de la Santé et de la Recherche Médicale (INSERM), Paris, France

**Keywords:** precision medicine, precision health, phenotype, genomics, individualized, population

Emerging scientific concepts set sail carried by the wings and tides of history, causing paradigm shifts, changing cultures, dissociating from old beliefs and promoting appropriate technological advancements that render them operable. The emerging concept of “Precision Medicine/health,” which has been hailed as the future of medicine for well over a decade is one such concept.

In this Research Topic, “*The path toward precision health prospects and challenges*,” nine articles reflect on key elements of the current practice of precision medicine, outlining triumphs as well as potential hurdles and gaps. Some of the most spectacular examples of these triumphs have been in pharmacogenomics. Visvikis-Siest et al., highlight in their article (Milestones in personalized medicine: from ancient time to nowadays-the provocation of COVID-19), examples of these, while emphasizing the dire want of personalized solutions and treatments tragically and ironically underscored by the SARS-CoV-2 pandemic. In fact, the pandemic blatantly exposed the feebleness of health systems that still rely on outdated concepts of “average” patients.

Although genomics was key in freeing science from the clutches of a “one gene one disease” set of mind, introducing us to realms of genomic interactions in the complex milieu of systems biology, science still largely grapples with these within one-dimensional perspectives of the biological world. Considerations of human-microbial host-parasite relationships thus remain beyond the purview of current genomic and transcriptomic approaches and consequently so too do potent modifiers of phenotypic outcomes resulting from complex scenarios in both infectious and non-infectious disease.

Moreover, although precision medicine owes much of its current utility and relative success to genomic advancements and the explosion of genomic databases, this progress does not come without cost. It is accompanied by profound ethical and societal questions, including those of “ownership” of personal or collective information and the labeling of groups and individuals with measured or reported phenotypes. Furthermore, the increased dependence on specific patient's genetic material lowers the value of knowledge based on “averaged individuals.” This use of patient and population specific genomic data to populate the public genetic databases that inform clinical and public health decisions raises, in addition to the above-mentioned questions of “ownership” of genetic data, concerns about the affordability of the technology for certain countries and individuals and thus the issue of equity in benefiting from these approaches.

The highlighted differences between groups and populations underscore the place of inclusiveness in research, publications and databases. Bibliometric and scientometric studies reveal the current scope, focus and state of precision medicine, its disparities and its trends and changes over time. Zhu and Zhang 's work on Emerging Trends and Research Foci in Cataract Genes gives us an example of such work.

Any platform for precision medicine must also include tools for translating relevant research into appropriate, applicable and evidence-based standards for clinical practice.

In “*Legal challenges in precision medicine: What duties arising from genetic and genomic testing does a physician owe to patients?”* McGrath et al., highlight the difficulties that specialist and non-specialist physicians face in understanding, communicating and utilizing genomic data, the challenges in establishing standards of practice in this rapidly changing domain and the ethical and, particularly, legal risks arising when utilizing (or failing to appropriately utilize) genomic technologies.

The spectrum of individualized phenotypes extends from the risk of exposures in healthy individuals through the vast spectrum of clinical phenotypes and up to death. Preventive precision medicine, with its applications and caveats, ideally aims to stratify individuals into finite and well-defined risk groups, incorporating biomarkers predictive of risk to guide pre-emptive interventions. In this collection, the death phenotype, as the ultimate phenotype to prevent, is the subject of two articles published by Chen et al., “*development of a simple risk model to predict mortality in patients with osteosarcoma of the extremity*” and by Primorac et al., “*sudden cardiac death-a new insight into potentially fatal genetic markers*.” Both, by employing genomic or clinical data, attempts with varying degrees of success to predict the risk of death. They reveal the challenges of identifying the underpinnings of extreme phenotypes, even when they are believed to be explainable by a single or few genomic changes. Multi-omic approaches, including epigenomics, transcriptomics, proteomics, metabolomics, and microbiomics, could come handy in refining such disease phenotypes, including death. Such a complex undertaking would require sophisticated tools such as artificial intelligence.

In conditions, classically, attributed to a single input, such as Mendelian diseases, heterogeneity in the causative input, together with genetic and environmental modifiers, can create complex and varied outcomes. It is therefore imperative to revisit paradigms and dictums, such as “one gene-one phenotype.”

Even for conditions where a single gene, or even a single mutation, is primarily responsible for disease initiation, significant and impactful variation in clinical phenotype, disease progression and response to therapy can arise from genetic, environmental or lifestyle modifiers.

In this collection, Yahia and Stevanin explore the “*History of gene hunting in hereditary spinocerebellar degeneration*,” highlighting the inherent genetic heterogeneity and reviewing the tools used in its elucidation. Ngo-Bitoungui et al., investigate the contribution of previously identified kidney Dysfunction-Related Gene Variants to kidney dysfunction in a cohort of Sickle Cell Disease Patients from Cameroon.

As for the common polygenic noninfectious diseases which occupy the widest space in the disease susceptibility spectrum, such as the phenotypically and genetically interrelated metabolic and cardiovascular diseases, genomics can become instrumental in redefining both their phenotypes and underlying molecular basis. Diabetes is one example that can benefit from such reclassification. The article by Fedotkina et al., (*Novel reclassification of adult diabetes is useful to distinguish stages of B-cell function linked to the risk of vascular complications: The DOLCE study from Northern Ukraine*) gives a glimpse of the utility that such approaches, which tend to greater precision in classification, can achieve.

There is no other place, where the complex interaction between genotype and phenotype as applied to precision medicine could be extensively elucidated and understood than the African continent. Africa is mankind's ancestral home and the store of its greatest wealth of genetic diversity. It has equally rich social and cultural legacies relevant to the understanding and practice of precision medicine. Hussein et al., (*individualized medicine in Africa: Bringing the practice into the realm of population heterogeneity*), present their views on the strengths and deficiencies of Precision Medicine in the African context, emphasizing challenges and opportunities of adopting such approaches in populations of high effective size and suggesting how population genomics could aid the transition between generic and personalized approaches.

Although precision medicine falls under the wider umbrella of precision health (which includes in addition to clinical medicine aspects of public health, community and preventive medicine, and health promotion,) the emphasis in much of the published literature (this topic included), is on clinical precision medicine. This reflects a rather global bias in understanding and managing issues and challenges of health and disease which we hope will become less pervasive as the precision health community grows and expands.

## Author contributions

All authors listed have made a substantial, direct, and intellectual contribution to the work and approved it for publication.

## Conflict of interest

The authors declare that the research was conducted in the absence of any commercial or financial relationships that could be construed as a potential conflict of interest.

## Publisher's note

All claims expressed in this article are solely those of the authors and do not necessarily represent those of their affiliated organizations, or those of the publisher, the editors and the reviewers. Any product that may be evaluated in this article, or claim that may be made by its manufacturer, is not guaranteed or endorsed by the publisher.

